# Rapid Decline of a Grassland System and Its Ecological and Conservation Implications

**DOI:** 10.1371/journal.pone.0008562

**Published:** 2010-01-06

**Authors:** Gerardo Ceballos, Ana Davidson, Rurik List, Jesús Pacheco, Patricia Manzano-Fischer, Georgina Santos-Barrera, Juan Cruzado

**Affiliations:** 1 Instituto de Ecología, Universidad Nacional Autónoma de México, México D.F., México; 2 Facultad de Ciencias, Universidad Nacional Autónoma de México, México D.F. México; Stanford University, United States of America

## Abstract

One of the most important conservation issues in ecology is the imperiled state of grassland ecosystems worldwide due to land conversion, desertification, and the loss of native populations and species. The Janos region of northwestern Mexico maintains one of the largest remaining black-tailed prairie dog (*Cynomys ludovicianus*) colony complexes in North America and supports a high diversity of threatened and endangered species. Yet, cattle grazing, agriculture, and drought have greatly impacted the region. We evaluated the impact of human activities on the Janos grasslands, comparing changes in the vertebrate community over the last two decades. Our results reveal profound, rapid changes in the Janos grassland community, demonstrating large declines in vertebrate abundance across all taxonomic groups. We also found that the 55,000 ha prairie dog colony complex has declined by 73% since 1988. The prairie dog complex has become increasingly fragmented, and their densities have shown a precipitous decline over the years, from an average density of 25 per ha in 1988 to 2 per ha in 2004. We demonstrated that prairie dogs strongly suppressed woody plant encroachment as well as created open grassland habitat by clearing woody vegetation, and found rapid invasion of shrubland once the prairie dogs disappeared from the grasslands. Comparison of grasslands and shrublands showed markedly different species compositions, with species richness being greatest when both habitats were considered together. Our data demonstrate the rapid decline of a grassland ecosystem, and documents the dramatic loss in biodiversity over a very short time period concomitant with anthropogenic grassland degradation and the decline of a keystone species.

## Introduction

Global environmental problems have become more acute as a consequence of ever-increasing pressures from human activities, resulting in an alarming loss of biological diversity. These losses are rapidly reducing Earth's life support systems and the services that nature provides, such as the clean air and water we all depend on [1], [2]. Grasslands have become one of the most imperiled ecosystems in the world, and are facing increasing threats by multiple anthropogenic activities. Their future depends greatly on the future of agriculture and grazing [3], [4]. Indeed, humans depend greatly on grasslands for overall food production, which is projected to increase by more than 75% over the next 30 years to support the projected doubling of the human population and its growing need for food [5]. Throughout the world, grasslands are being converted either to croplands or desertified shrublands from overgrazing by livestock [6], [7]. The loss and fragmentation of grasslands is causing the extinction of uncounted populations and species, changes in the structure and function of ecosystems, depletion of environmental services, and decline in human well-being [e.g.], [7], [8,9].

Only around 20% of North America's central grasslands have not yet been developed or converted to cropland, and much of what remains is utilized for cattle grazing [7], [10]. Widespread desertification of North America's semi-arid grasslands has already occurred [11], [12], [13], [14]. Overgrazing results in the removal of perennial grasses and leads to shrub invasion, with the result that these grasslands have been replaced by desert shrub communities often dominated by unpalatable plants such as ephedra (long-leaf jointfir, *Ephedra trifurca*), and palatable ones such as mesquite (*Prosopis glandulosa*) whose seeds are readily eaten by cattle [6], [11], [14]. In some regions, overgrazing has resulted in the widespread replacement of perennial grasses by forbs and annual grasses [15]. Perennial grasslands are characterized by relatively stable grass cover, uniform distribution of available resources, and stable soils. In contrast, desertified grasslands dominated by annual grasses have more temporally variable vegetation and resources, and are subject to severe soil erosion [11], [16], [17].

The semi-arid grasslands of the Janos region of northern Chihuahua, Mexico, have been subject to intensive cattle grazing and some exceptionally dry periods over the last decade, providing an ideal opportunity to study the ecological consequences of grassland degradation. These grasslands support one of the largest remaining black-tailed prairie dog (*Cynomys ludovicianus*) complexes on the continent (14,796 ha), and the only significant complex remaining in the semi-arid grassland system of the American Southwest/northern Mexico region [15]. Prairie dogs are fossorial rodents that live in large colonies and are considered to be keystone species and ecosystem engineers, as they transform grassland ecosystems through their burrowing and herbivory [18], [19], [20]. They create important habitats for other animals and are key prey for many predators, increasing heterogeneity and biodiversity in grasslands [19], [21], [22], [23], [24], [25]. Prairie dogs also play an important role in preventing shrub invasion by consuming the seeds and seedlings of shrubs, in turn helping to maintain grassland ecosystems [26].

While anthropogenic activities are transforming the globe, few studies have documented the effects of the decline of a grassland ecosystem due to human activities and the consequential effects on plant and animal communities. Here, we document the effects of the rapid decline of a grassland ecosystem on vertebrate biodiversity in the Janos region. We also assess human land use changes and long-term weather (i.e., precipitation) to better understand the large-scale factors driving the observed changes over time. Little is known about how unique these prairie dog grassland communities are, how fast land degradation can impact biodiversity, and what the main conservation lessons are. Specifically, we address the following questions: i) Have there been large landscape-scale changes in area covered by native plant communities, concomitant with intensive human land use (i.e., grazing and agriculture)? ii) If so, have those changes affected the area covered by the prairie dog complex in the same period? iii) Do prairie dogs reduce the invasion of shrubland and thus promote the maintenance of semi-arid grasslands? iv) Do grasslands and shrublands differ in vertebrate biodiversity, and if so, what is their combined contribution to regional biodiversity? v) Have there been concomitant declines in vertebrate diversity parallel to the decline in prairie dog colonies over a short-term (i.e., ca. 10-year) period? Finally, we discuss specific conservation strategies to restore the prairie dog colonies and the grassland ecosystem.

## Results

### Landscape scale changes over time

**Figure 1:**
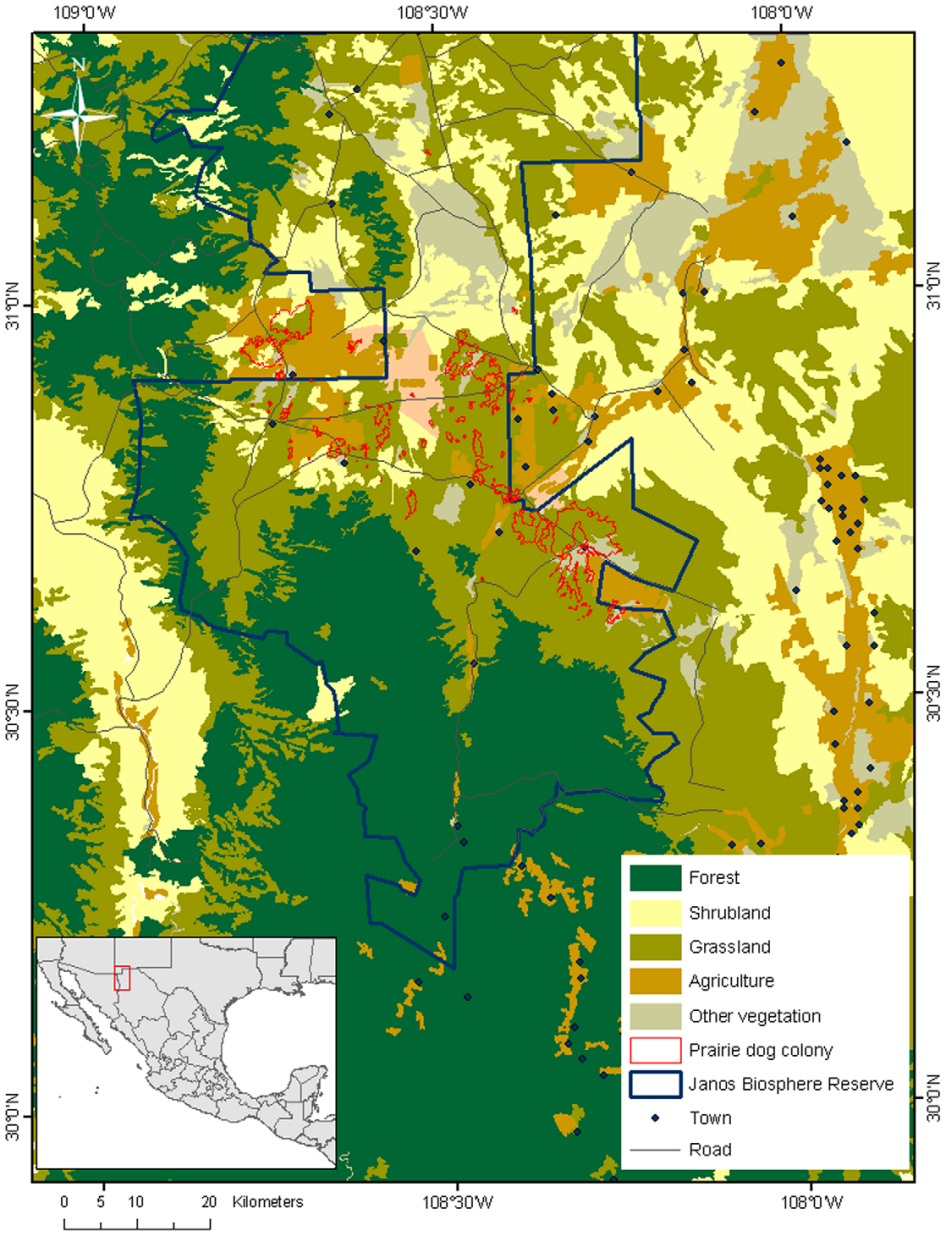
Location of the Janos region showing current land uses and natural vegetation cover in 2005. The Janos region covers 1 millon hectares in northern Mexico, bordering the United States. The main plant communities are grasslands, shrublands, and temperate forests. This region still maintains one of the largest prairie dog complexes in the world, and the Janos Biosphere Reserve has been designated to protect this biologically important region.

**Figure 2:**
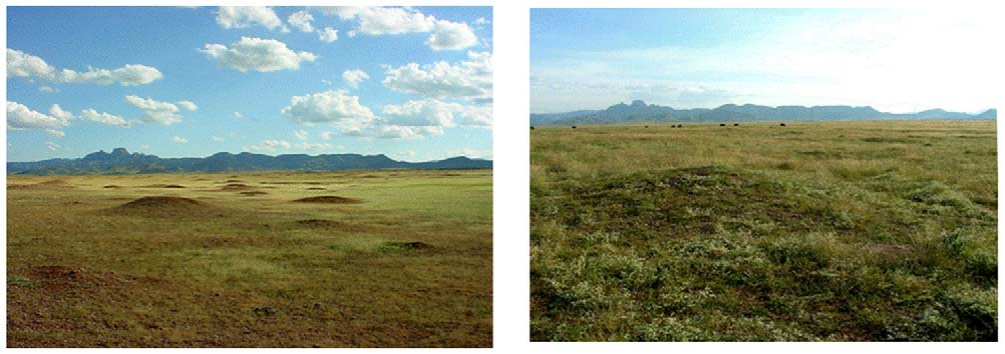
Changes in the El Cuervo prairie dog colony in 2002 (left), and of the same area in 2006 (right). These photos show the rapid loss of prairie dogs within the largest colony of the Janos grasslands, following two decades of intensive land use and drought. Note the sparse coverage of annual grasses and forbs and the lack of perennial grasses, which is characteristic of degraded grasslands in Janos. These plants are only available during the rainy season and most of the year the area is bare ground.

The Janos region covers 1 million ha of grasslands, shrublands, and mountain plant communities (Figure 1). We observed anthropogenic degradation of the native vegetation over the course of our research, leading to surprisingly extensive and rapid changes, especially those due to overgrazing and intensive agriculture. In only a decade, from 1990 to 2000, around 6% (46,493 ha) of the grasslands were completely transformed, and there also was a large, 142-fold increase (from 6,645 to 52,123 ha) in bare ground cover, in areas that used to have grasslands. Similarly, the area used for intensive agriculture, especially by center-pivot crop fields, showed a 1,757-fold (731 ha to 12,845 ha) increase from 1993 to 2008, all of which were plowed in either prairie dog colonies or native grasslands. Compounding the impacts of intensive land use, the region experienced a prolonged drought, with a period of below average precipitation between 1993–1996 and 1998–2003. The years from 1993–2005 represent the driest period in the last 50 years (Figure S1). The effects of drought were very severe when coupled with overgrazing, and were one of the leading causes of the conversion of grasslands to bare ground. For example, the communal grasslands of Ejido Casa de Janos, supported the largest prairie dog colony in Janos in 1993 (35,000 ha). The cattle grazing carrying capacity of this land was estimated at 200 head of cattle (M. Rollo, personal communication). However, they had 2000 animals during most of the time from 1993 to 2005. By 2005, most of the former prairie dog colony was abandoned with very few prairie dogs remaining (Figure 2).

### Prairie dog decline and shrubland expansion

**Figure 3:**
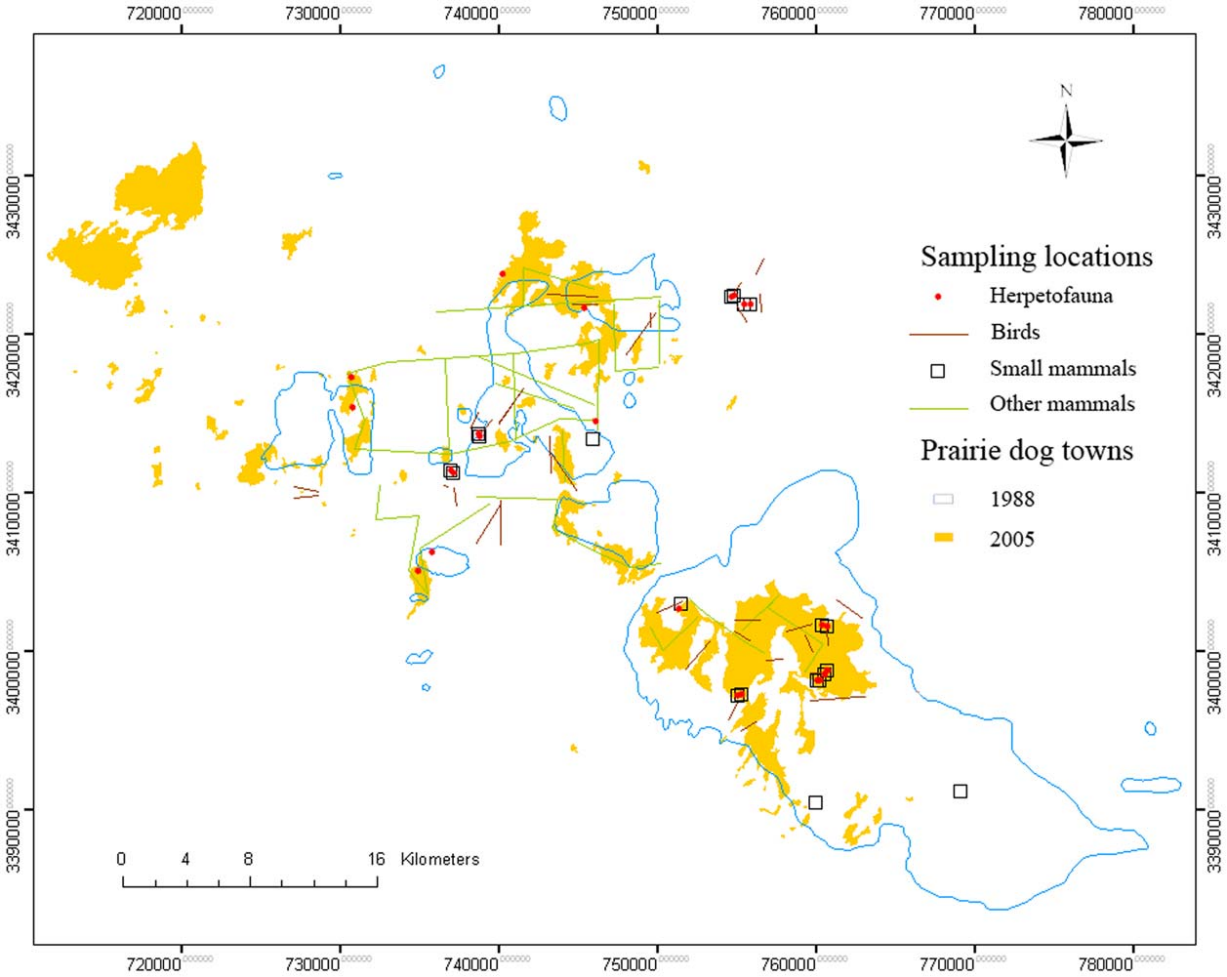
Change in area of the Janos-Casas Grandes prairie dog complex from 1988 to 2005. Extent of prairie dog colonies in 1988 and 2005, and location of the sampling sites 1992–2004: pitfall trap grids and transects for herpetofauna, point count transects for birds, Sherman trap grids for small mammals, and spotlighting transects for medium and large mammals (see Table A3 for specific sampling periods for each group).

The decline of the natural vegetation at a landscape level in Janos had a severe impact on the prairie dog colony complex. The 55,000 hectares of grassland occupied by prairie dogs in 1988 experienced a 73% decline by 2005, representing a loss of around 40,000 ha (Figure 2 and 3). The original eight colonies, with an average size of 6,250 ha, were converted into more and smaller (437 ha on average) colonies (31). The remaining colonies were scattered along the original geographic distribution area. A few very large colonies still remain such as El Cuervo (6,300 ha) and Monte Verde (3,250 ha).

**Figure 4:**
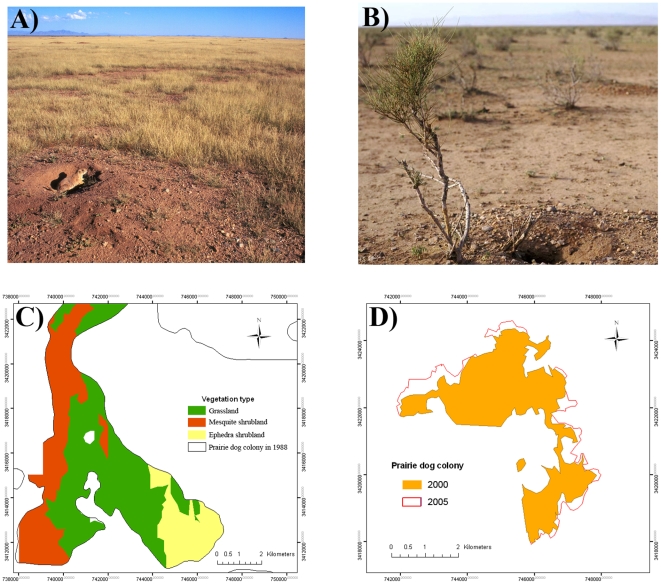
Effect of prairie dog presence/absence on maintenance of the grassland ecosystem. A) Prairie dog colony in Janos, Chihuahua, Mexico, note the absence of woody plants. B) Prairie dog expansion into ephedra shrubland by physically damaging the invading shrubs. Visible in the picture is the extensive browsing of shrubs, and several burrows at the base of the shrub which exposes the roots (see also map in Fig. 2D). C) Polygon of the southern portion of the prairie dog colony of Los Ratones, which was covered by grassland in 1988, showing a 43% advance of honey mesquite (Prosopis grandulosa) and ephedra (Ephedra trifurca) shrubland after the colony was poisoned between 1988 and 1990. D) Sixteen percent expansion of the La Báscula prairie dog colony into ephedra shrubland between 2000 and 2005.

The loss of prairie dogs had profound negative impacts on the maintenance of grasslands and the expansion of shrublands. We documented major changes in the grassland – shrubland landscape composition, related in part to the presence of prairie dogs that strongly suppress woody plant encroachment and create open habitat through their foraging and clipping behavior (Figures 4A and B). Here, we took advantage of an (un-)natural experiment where prairie dogs had been poisoned or had recolonized the landscape, providing us the opportunity to assess changes in grassland and mesquite cover in relation to the presence or absence of prairie dogs. In one case, prairie dogs were poisoned in the 1,588 ha Los Ratones colony between 1988 and 1990. The effects of prairie dog removal on the grassland were already visible by 1996. Thirty four percent (1,653 ha) of the previous open grassland was invaded by either mesquite (14%, 693 ha) or ephedra (20%, 960 ha) shrubland in just eight years (Figure 4C). In the second case, between 2000–2005, the La Bascula prairie dog colony expanded 16% (208 ha/1,270 ha) into closed ephedra shrubland through the physical removal of shrubs (Figure 4D). Additionally, ephedra shrubs were 55% shorter (34.1 cm vs 75.5 cm) in the prairie dog colony, and 81% of them had signs of prairie dog clipping; in comparison, ephedra shrubs only 50 m away from the colony were taller and only 3% had signs of clipping (Figure 4B, Table S1). The edge of the colony advanced up to 546 m into the shrubland during this five-year period, converting the area back into an open grassland habitat. In summary, our data clearly indicates the effects of prairie dogs in maintaining the presence of open grasslands and limiting the expansion of shrubland.

### Comparison of grassland and shrubland vertebrate communities

**Figure 5:**
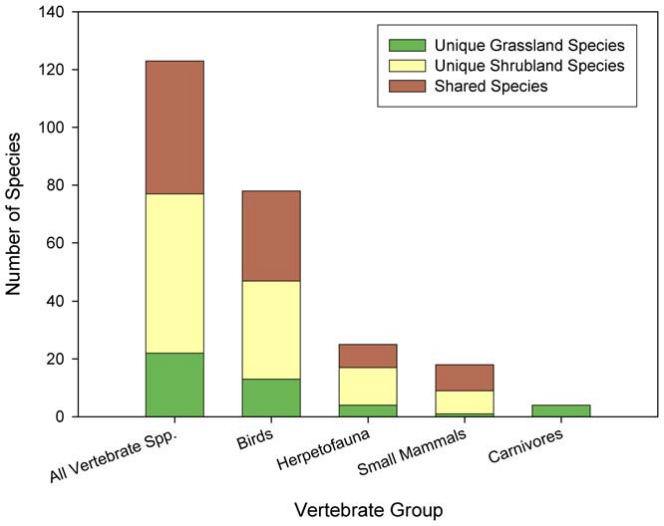
Vertebrate species richness in grassland and shrubland habitats. Total number of shared and unique species of all vertebrate groups combined, birds, small mammals (including lagomorphs), herpetofauna, and carnivores on the grassland and shrubland habitats over all sample periods (1994, 1995, 1996, 2000, 2001, 2002, 2003, and 2004; see Table A3 for specific sampling periods for each group).

**Figure 6:**
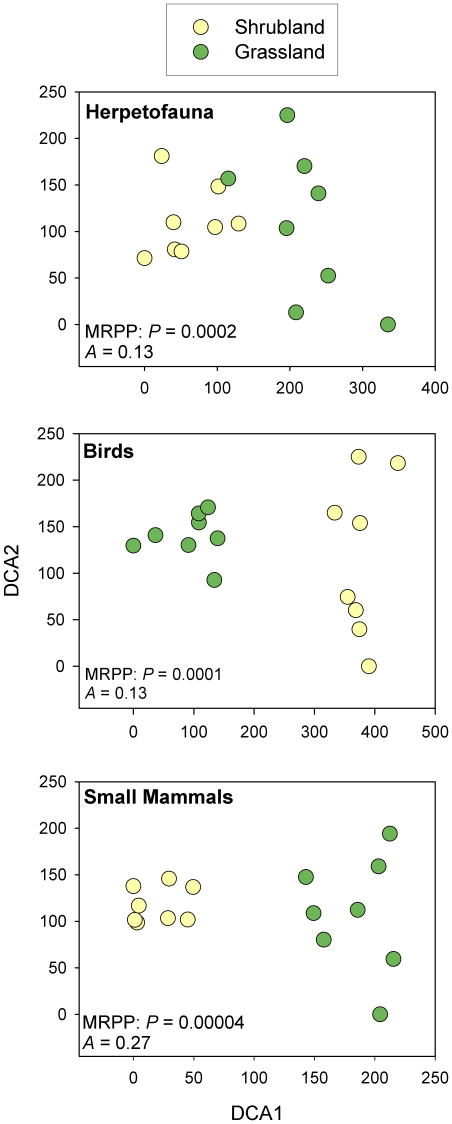
Total abundance of vertebrate species in grassland and shrubland habitats. Total abundance of herpetofauna, birds, small mammals, rabbits, and carnivores on the prairie dog grassland and shrubland habitats over all sample periods (1994, 1995, 1996, 2000, 2001, 2002; N = 8 for each sample period). Asterisks (*) indicate significant differences in abundance between the habiats at P<0.01.

**Figure 7:**
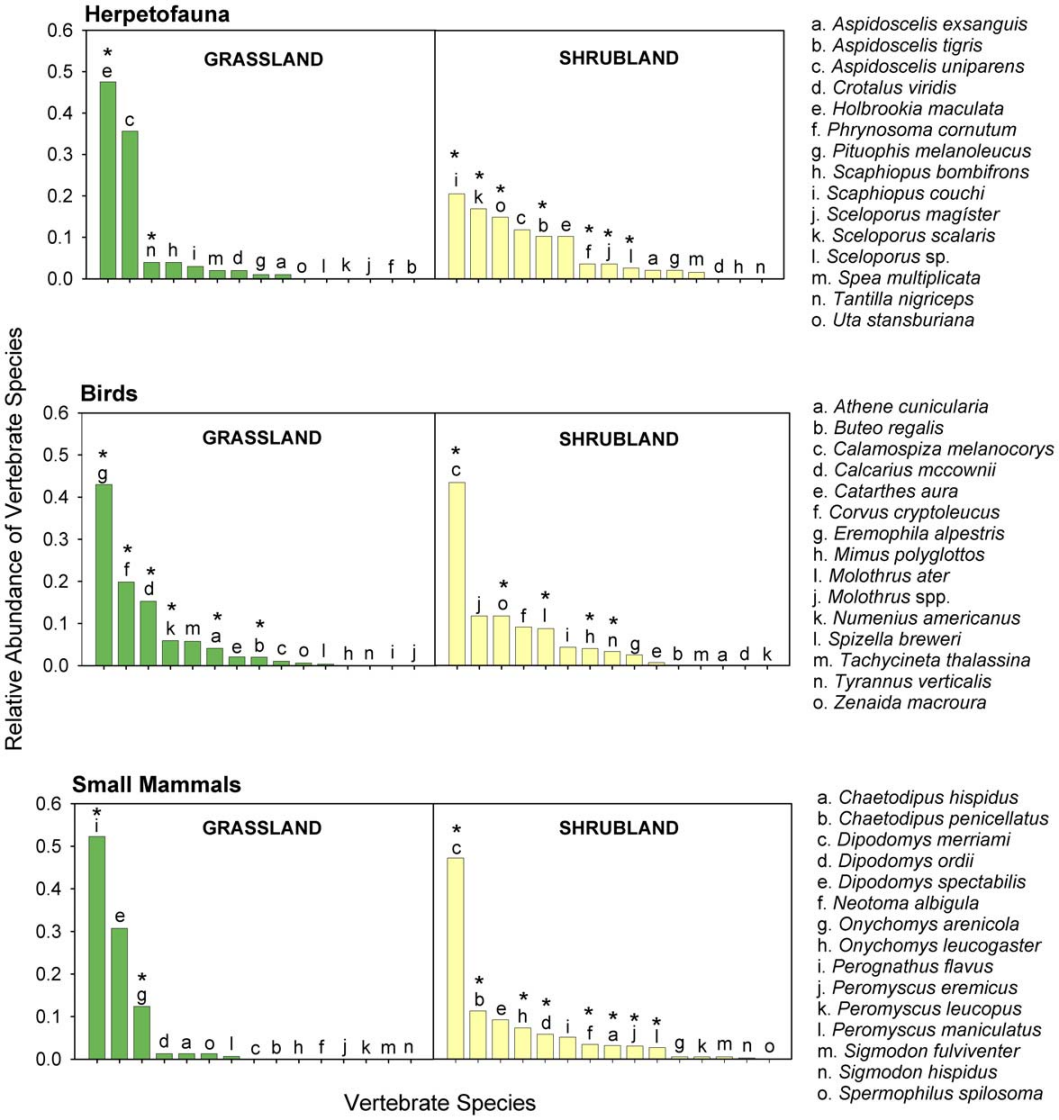
Differences in species composition between grassland and shrubland habitats. Detrended Correspondence Analysis (DCA) ordinations based on species composition of herpetofauna, birds, and small mammals. Multi-Response Permutation Procedure (MRPP) demonstrates that the species compositions of these vertebrate groups is significantly different between the prairie dog grasslands and the shrublands (1994, 1995, 1996, 2000, 2001, 2002; N = 8 for each sample period).

The prairie dog colony grasslands and the shrublands had differences in vertebrate species richness, composition, and abundance in the four vertebrate classes evaluated. As we predicted, species richness was greater when both habitats were considered together, but shrublands had greater species diversity across all vertebrate classes, except large carnivores (Figure 5). However, simply comparing species richness to assess differences can be misleading, so we compared species composition and found that the two plant communities exhibited markedly different compositions, with many species being unique to each habitat type (Figures 5, 6 and 7, Table S2). Indeed, herpetofauna, birds, and small mammal assemblages differed strongly between the two plant communities, when analysed with both a Detrended Correspondence Analysis (DCA) and a Multi-Response Permutation Procedure (MRPP) (*P*<0.0002, for all tests) (Figure 6).

**Figure 8:**
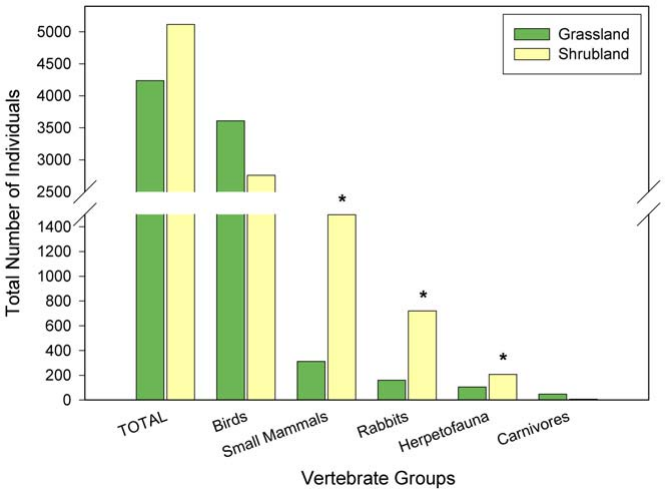
Relative abundance of vertebrate species in grassland and shrubland habitats. Relative abundance of herpetofauna, bird, and small mammal species on the prairie dog colony grasslands and the shrublands over all sample periods (1994, 1995, 1996, 2000, 2001, 2002; N = 8 for each sample period). Asterisks (*) indicate significant differences in abundance between the habitats at P<0.05.

Large differences in relative abundance and dominance of vertebrate species also were present between the two habitats (Figures 7 and 8). While total vertebrate species richness and abundance were greater in shrublands, there was considerable variation among the vertebrate groups (Figure 8). Total abundance of birds did not differ significantly between the two habitat types. Yet, birds were almost twice as species rich in the shrubland than in the grassland habitat (W: *C* = 38.0, *P* = 0.0037), and of the 24 different families of birds observed, 20 showed significant differences in abundance between the two habitat types (W: *P*<0.05, for all tests). At a species level, grasslands were dominated by Horned Larks (*Eremophila alpestris*) and Chihuahuan Ravens (*Corvus cryptoleucus*), while shrublands were dominated by Lark Buntings (*Calamospiza melanocorys*) and Mourning Doves (*Zenaida macroura*) (Figure 7 and Table S2).

Species richness of small mammals and total abundance of both small mammals and lagomorphs were significantly higher in shrublands than grasslands (species richness W: *C* = 36.0, *P* = 0.0002; abundance W: *C* = 36.0, *P* = 0.0008; Chi-Square: *x* ^2^ = 20, df = 7, *P* = 0.007, respectively; Figure 5, 7, and 8). Most species and all trophic and family groups of small mammals were significantly more abundant in the shrubland habitat (W: *P*<0.01, for all tests; Figure 7 and Table S2). Silky pocket mice (*Perognathus flavus*) and Mearn's grasshopper mice (*Onychomys arenicola*) showed a strong association with grasslands, while Merriam's kangaroo rats (*Dipodomys merriami*) were more associated with the shrublands. Of the two lagomorph species, the black-tailed jackrabbits (*Lepus californicus*) were more than ten-times more abundant in the shrublands than in the grasslands (Chi-Square: *x* ^2^ = 22, df = 7, *P* = 0.003), while the desert cottontails (*Sylvilagus audubonii*) did not show differences.

Abundance (*N* = 52) and richness (*N* = 5) of carnivores were greater in the grasslands than in the shrublands (abundance *N* = 8, richness *N* = 1; Figures 7, and 8). The relatively higher number of carnivore species observed in the grasslands versus the shrublands could have been influenced by the greater sampling visibility in the grasslands versus the shrublands, but Program Distance adjusts for sighting distance making the overall effect of visibility minor. Trends among species were somewhat varied (Figure 7 and Table S2). For example, the relative density of coyotes (*Canis latrans*) was greater in the shrubland habitat (*D* = 0.002) than in the grassland (*D* = 0.001), but kit foxes (*Vulpes macrotis*), skunks (*Mephitis* spp.), and badgers (*Taxidea taxus*) all had higher densities in the grasslands (*D* = 0.014, *D* = 0.001, *D* = 0.1, respectively) than in the shrublands (*D* = 0, for all three species). One black-footed ferret (*Mustela nigripes*) also was observed in the grassland where they were reintroduced in 2001.

As expected, reptiles and amphibians were more diverse in the shrublands when compared to the grasslands, with two-fold increases in both species richness (W: *C* = 42.5, *P* = 0.009) and abundance (W: *C* = 46.5, *P* = 0.02; Figure 5 and 7). Toads and lizards were significantly more associated with shrublands than grasslands (W: *C* = 42.5, *P* = 0.009; *C* = 48.0, *P* = 0.03, respectively; Figure 7 and Table S2).

Importantly, the prairie dog grasslands harbored many more priority conservation species compared to the shrublands. There were more endangered, threatened, and/or keystone species, and in larger numbers, in the grasslands as compared to the shrublands, including Burrowing Owls (*Athene cunicularia*), Ferruginous Hawks (*Buteo regalis*), Bald Eagles (*Haliaeetus leucocephalus*), Curlews (*Numenius americanus*), kit foxes and black-footed ferrets (Figure 7, Table S2).

### Temporal variation in vertebrate community diversity

**Figure 9:**
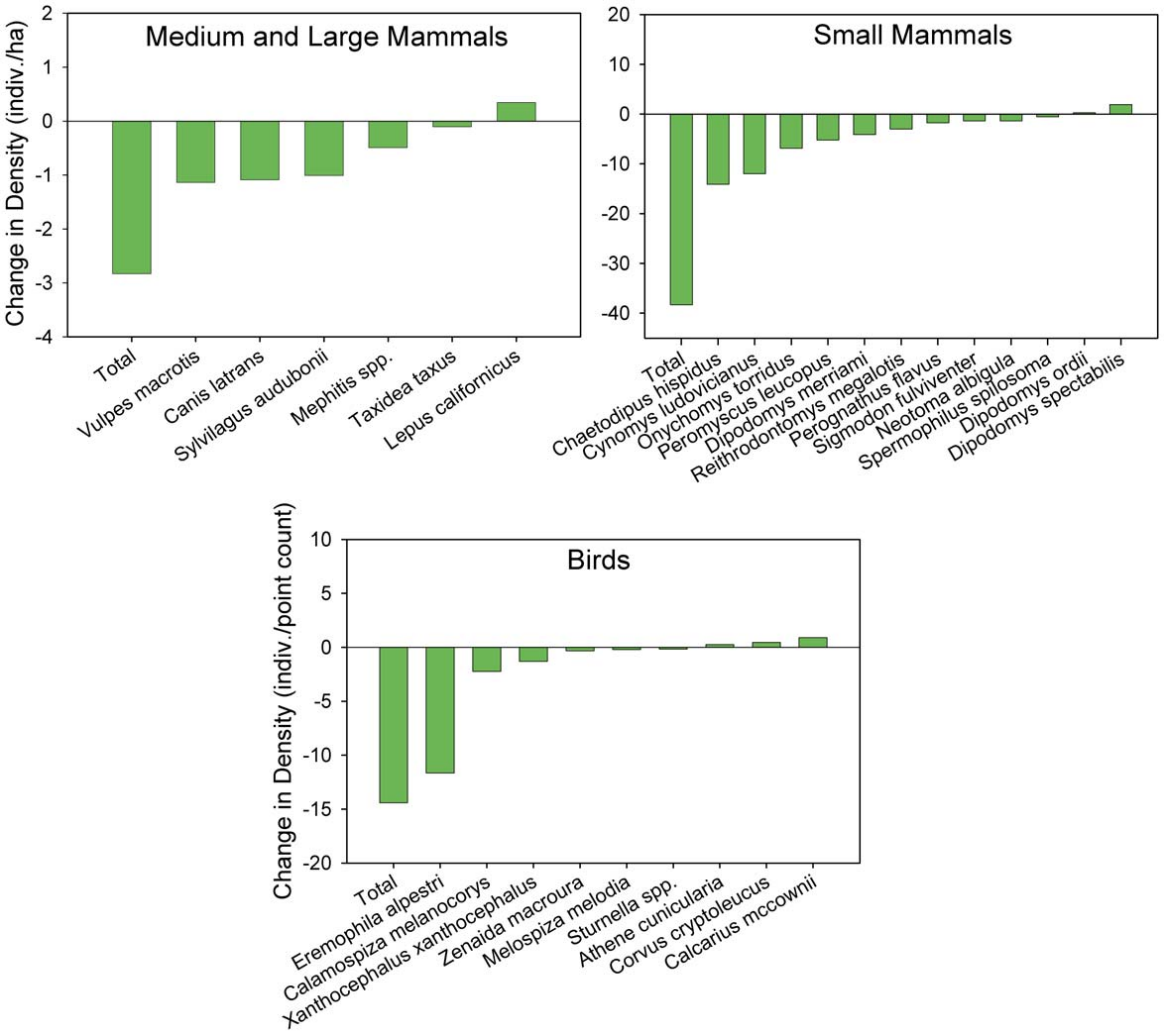
Decline in vertebrate species density over a ca. 10-year period (1992–2004). Mammal and bird species in the Janos prairie dog grasslands showing dramatic declines in densities over time. (Note prairie dog densities are compared from 1994–2004.) Of the 33 bird species that were sampled, only those that exhibited a 2-fold or greater change over time are shown here.

To assess whether the comparisons of current vertebrate diversity in the Janos region were the outcome of the land use changes that have occurred in the last two decades, we compared the vertebrate species assemblages in Janos over a ca. 10-year period. The results indicate that the vertebrate community structure and diversity has showed a much more complex, dynamic scenario than when only comparing current diversity data. In the absence of experimental data we cannot determine specific causes of these changes with certainty, but there was a pervasive correspondence among land use changes and vertebrate community changes. Dramatic declines have occurred in both grassland birds and mammals over the ca. 10-year period concomitant with the large declines in area covered by grasslands (Figure 9). Birds showed a two-fold decline in density from 1994 to 2004. Unsurprisingly, species most typical of grasslands showed the largest declines, such as Horned Larks that experienced four-fold declines. Cottontail rabbits and small mammals also showed large declines with their densities decreasing by more than 50%. Prairie dogs exhibited the second greatest decline among small mammals with an 8-fold decrease in density just over the course of this study, from 16 per ha in 1994 to 2/ha in 2004. Medium and large mammals showed an overall 12-fold decline in abundance, ranging from more than a 20-fold decline in coyotes and skunks, to an eight-fold decline in the threatened kit foxes, and a five-fold decline in badgers.

## Discussion

Our results reveal profound, rapid changes in the Janos grassland ecosystem. We documented large declines in the distribution of prairie dog colonies as well as in vertebrate abundance across all taxonomic groups evaluated, concomitant with severe land degradation. Further, the grassland and mesquite shrubland provided habitats for two markedly different vertebrate communities. Herpetofauna, mammals, and birds all differed greatly in community structure between the two habitats, and many were unique to only one of the habitat types. A few decades ago, the Janos region was a mosaic of grasslands mostly occupied by prairie dog colonies, shrublands, and riparian vegetation [27]. Under these rapid environmental changes, the grasslands are being transformed to shrublands, leading to desertification and biodiversity loss, as has been shown in other regions of the southwestern United States [28]. The transformation of the grasslands is clearly linked to prairie dog loss and intensive land use practices, exacerbated by drought.

The results follow the broader trend of over 95% decline in prairie dog populations throughout their range [29]. The decline in prairie dogs is known to have cascading effects on other animals, as many species associate with the open habitats and burrows that prairie dogs create and depend on prairie dogs as prey, such as black-footed ferrets, Mountain Plovers (*Charadrius montanus*), kit foxes, coyotes, badgers, raptors, and Burrowing Owls [e.g.], [8], [23], [30], [31,32]. As such, the decline in prairie dogs has likely contributed to the overall decline in the vertebrate community observed in our study. Our research also demonstrates that prairie dogs engineer grasslands by clipping shrubs, converting an invading shrubland back to open grassland. Conversely, the consequence of their loss results in shrub invasion. These results are consistent with Weltzin et al. [26] who found that prairie dogs eat the seeds and seedlings of mesquite shrubs and that shrub establishment occurs following their removal. They also surmised that the mass extermination efforts designed to eliminate prairie dogs over the last century in the United States likely contributed to the widespread expansion of mesquite shrubland.

Differences in vertebrate community structure between the prairie dog grasslands and the shrublands were expected. Yet, the differences we found were unexpected when compared to previous research in the region as well as reported ecological associations between species and their habitats. For example, although the mesquite shrublands were richer in bird species than the prairie dog colony grasslands, as would be predicted for structurally more complex habitats [33], [34], [35], this result was the opposite from previous research in the area where bird species were three-times more rich in the grasslands than in the shrublands [36]. Another example is the bunchgrass lizard (*Sceloporus scalaris*), a species highly associated with perennial grassland habitat [37], but which was more abundant in the shrublands in our study. This species may have preferred the more structurally complex shrublands compared to the heavily grazed grasslands, as it has been found to be ten-times more abundant in ungrazed perennial grassland than grazed grassland [38]. Even some small mammals common to grasslands, such as Ord's kangaroo rats (*D. ordii*) and hispid pocket mice (*Chaetodipus hispidus*), were significantly more common in the shrublands in our study.

These unexpected results and the rapid, large declines in grassland vertebrates are undoubtedly related to the overall grassland deterioration. After years of below-average precipitation and continuous overgrazing by domestic cattle, the Janos grasslands have become desertified annual grasslands and shrublands with extensive areas of bare ground [39], [40]. In this degraded system, productivity is reduced and important resources, like food and refuge, are scarcer and less dependable. Indeed, grassland productivity declined in the Janos region from a 0.162 Normalized Difference Vegetation Index (NDVI) in 1990 to 0.068 in 2000 [39]. Cattle management practices in the region have not adapted to the productivity change, and much of the land is communally owned, referred to as Ejido lands, which has resulted in the tragedy of the commons [41]. Shrubs have expanded not only in Janos, but throughout the Chihuahuan Desert grassland since the late 1800's, a consequence of livestock grazing and seed dispersal, the disruption of fire regimes, and drought conditions [42], [43], [44], [45].

The contraction of prairie dog colonies between 1988 and 1996 was due to poisoning, and between 1996 and 2005 was a consequence of the synergistic effect of drought and overgrazing as well as increasing land conversion to agriculture primed by the expansion of utility lines [46], [47]. While we only report the change in prairie dog colony sizes from 1988 and 2005, intermediate mapping efforts in 1996 and 2000 showed that the loss of prairie dog colonies was a continuous process [46], [48]. To date, no plague events, which have been the cause of prairie dog declines elsewhere, have been recorded in the Janos prairie dog complex. Our findings on the decline of the grassland system in Janos are likely taking place in other areas of the Chihuahuan Desert and even in other drylands of the world [6], [11], [49]. The implication of the rapid loss in biodiversity to the overall conservation of grassland systems is dramatic, as grasslands cover about 40% of the planet's land surface and provide a large proportion of the world's food supply [7], [12]. Such losses in biodiversity impact the provision of ecosystem services that grasslands provide. Indeed, as shown by our data, the loss of prairie dogs and inadequate land management practices are resulting in shrub encroachment, reducing the capability of the grasslands to provide forage for cattle, carbon sequestration, soil stability, water infiltration, and other ecosystem services. Our study provides evidence of the ecological decline of an ecosystem, as a consequence of the loss of a keystone species and extreme environmental pressures imposed by overgrazing, intensive agriculture, and drought. Global warming is further predicted to increase the frequency of droughts and aridity of the Chihuahuan Desert, causing up to 40% species turnover by 2055 [50], which we can expect to exacerbate the current ecological conditions in the Janos region, unless major changes in land management are made.

### Conservation implications

The rapid deterioration of the Janos grassland ecosystem has led us to propose conservation and management solutions that can be applied at a landscape level [14]. To do this, we designed a half million hectare biosphere reserve in the Janos region to help conserve the grasslands, prairie dogs, and regional biodiversity in a way that is compatible with human economic activities, especially grazing and agriculture (Figure 1). The Janos Biosphere Reserve has now been announced in the Official Registry of the Federal Government of Mexico and will become official later in 2009.

We are now working on a new paradigm in conservation for the Janos region that incorporates human activities as part of a large-scale conservation strategy, and avoids fighting the powerful cattle and agricultural industries to instead use them at a local scale as agents of restoration. We are designing management plans for using grazing and agriculture to maintain the grassland. For example, we are using cattle grazing to open grasslands and allow prairie dogs to re-colonize them more rapidly, and in turn helping the long-term maintenance of the grassland ecosystem. Reducing grazing pressure can allow grasses to grow and help restore the now absent fire. Fire and prairie dogs can limit shrubland encroachment and expand the more productive grasslands for the benefit of cattle ranching, and cattle and fire can reduce vegetation height that allows prairie dog colonies to expand into the grassland.

Similarly, we plan to use intensive agriculture to restore grassland that has been desertified to shrubland. Eliminating mesquite shrubland is extremely expensive, and therefore, usually impossible to do as a large-scale restoration approach. In Janos, there are thousands of hectares of such shrublands that can be reconverted to grasslands if industrial agriculture is first used to clear the invasive mesquite and plant commercial crops for a set period. After that period, these areas will be planted with perennial native grasses and converted into grasslands. There are incentives for both intensive agriculture and conservation to employ such a strategy. On one hand, the land for new agricultural fields is now limited because of the reserve, so using it as a restoration technique will allow the generation of a local income through agriculture to continue in the coming decades. On the other hand, using the economic power of agricultural groups to eliminate the invasive mesquite shrubs, a now impractical conservation strategy because of the cost, will be a major achievement for the long-term ecological restoration of the region. These are just a sample of the many possible human-ecological strategies that could be employed to restore and maintain native ecosystems in the Janos area and elsewhere.

Ultimately, for sound, long-term ecosystem conservation in the face of increasing global challenges, it is urgent to complement traditional land use and endangered species conservation approaches with novel strategies that couple the human dimension (e.g., culture) and ecological systems. As scientists, this is one of the most critical challenges of our time, and our responsibility includes finding answers for the problems.

## Methods

### Study Site

This study was conducted in the Janos Casas Grandes prairie dog complex, a mosaic of native grasslands and shrublands in northwestern Chihuahua, Mexico (30° 50′N, 108° 24′W) (Figure 1). The grasslands are presently dominated by the annual grasses, sixweeks threeawn (*Aristida adscensionis*), needle grama (*Bouteloua aristidoides*), and sixweeks grama (*B. barbata*), and numerous forbs. Perennial grasses present include poverty threeawn (*A. divaricata*), ear muhly (*Muhlenbergia arenacea*), burrograss (*Scleropogon brevifolius*), vine mesquite (*Panicum obtusum*), tobosagrass (*Pleuraphis mutica*), blue grama (*B. gracilis*), black grama (*B. eriopoda*), and red grama (*B. trifida*). The shrublands are dominated by mesquite, ephedra, and cholla (*Opuntia imbricata*). The climate is semi-arid, with hot summers and cold winters (*X* = 15.7°C, *range* = −12 to 50°C). Most of the precipitation occurs during the summer, with an average annual rainfall of 287 mm, although during most of our study rainfall was below average (1993–1996 and 1998–2003, Figure S1).

### Landscape-scale changes over time

To determine the large-scale vegetation changes over time in the Janos region, a supervised classification of satellite imagery (Landsat TM for 1990 and Lansat ETM+ for 2000) at a 25 m×25 m resolution, using ENVI software (ITT Visual Information Solutions v. 4.5) was made, using the resulting algorithms from a decision tree generated by the program See5 for Windows (Rulequest Research Data Mining Tools) [39]. The area of prairie dog colonies transformed to agricultural land was determined by counting the number of center pivot crops and measuring their diameter to determine area from a real color satellite image from 1993 [51] and from Google Earth in 2008 [52], over a 4,250 km^2^ area.

### Prairie dog decline and shrub control

To assess the change in area occupied by prairie dogs, the entire Janos prairie dog colony complex was mapped by following the contour of each colony in 1988 on horseback, walking, or flying, using a theodolite and topographic maps (1∶250 000). The complex was mapped again in 2005 by walking, cycling, or riding an ATV and taking coordinates with a Global Positioning System receiver (GPS) every 150 m. A prairie dog burrow was considered as being part of the same colony if it was <150 m from a previously mapped burrow, which represents roughly 1.5 times the distance prairie dogs have been observed moving away from their burrows in their foraging activities in the area.

Using the above methods, we followed parts of two colonies over time to evaluate the change in shrub expansion/contraction in response to prairie dog removal/colonization. One of the colonies originally mapped in 1988 (4,930 ha) was poisoned from 1988–1990, and the vegetation types on 2,500 ha of this colony were mapped in 1996 as part of another study [31], [46], which allowed us to document the expansion of shrubs into the area originally occupied by prairie dogs. Prairie dogs were observed building burrows and chewing ephedra at the edge of prairie dog colonies that were surrounded by shrublands. In one particular colony, mapped in 2000 (1,270 ha), the expansion was evident, so it was re-mapped in 2005 to document this expansion. To determine the impact of prairie dogs on ephedra shrubs, 100 individual shrubs were examined along a transect at the edge of the colony where it was expanding, and along an adjacent transect extending 50 m away from the colony. The height of each shrub was measured, and it was examined for presence or absence of prairie dog teeth signs, exposure of the roots by prairie dog burrows, and clipping of stems and branches.

Prairie dog densities were determined in 1994 (420 transects), 1995 (384 transects), 1996 (304 transects), and 1999 (234 transects) by counting the number of active and inactive burrows along 1 km×3 m wide transects within the prairie dog colony grasslands following methods described in Biggins et al. [53]. Parallel transects were established, each separated by 40 m. To reduce the observer error in assigning burrows as active or inactive, or other biases of the method [54], density estimates in 2001 onwards were based on maximum number of prairie dogs aboveground at any one time observed from 12 4.5 ha quadrants (triangles). The triangle method consisted of three triangles measuring 150 m per side (4.5 ha) around an observer located at the center. The vortices of the triangles were 50 m from the observer, so by turning around, the observer could record the number of prairie dogs within each triangle every 10 minutes.

### Comparison of grassland and shrubland vertebrate communities

Vertebrate diversity in grasslands with prairie dog colonies and in mesquite shrubland was assessed by establishing 4 replicate plots in grassland habitat and 4 replicate plots in shrubland habitat within a mosaic of a ca. 15,000 ha prairie dog colony and mesquite shrubland (Figure 2). Ten field trips, covering all 4 seasons, were conducted for all vertebrate groups between November 2000 and June 2002 (Table S3). While our previous studies have compared vertebrate diversity in prairie dog colonies and adjacent grasslands in this area between 1992–1996 [21], [55], [56], the grassland without prairie dogs is a transient vegetation type. It often is rapidly invaded by woody plants, mainly mesquite and ephedra, converting it into shrubland. Thus, our objective was to determine if the overall vertebrate diversity would decline when grasslands become desertified shrublands, losing species exclusive to the grasslands/prairie dog colonies [45], [57]. Reptile and amphibian diversity was compared with pitfall traps [58]. From 2000–2004, pitfall traps were established in a 3×3 grid array, with traps separated by 30 m in each of the 4 grassland study sites and 4 shrubland study sites (overlapping with the mammal grids and bird surveys during the same period, Figure 2, Table S3). Each trap consisted of a standard 20 liter plastic bucket [59]. The traps remained opened for three consecutive days in each sampling period. Each site was checked every morning or twice a day on extremely hot days.

Bird diversity was assessed with point count transects [60] from 1994–1995 in various colonies of the prairie dog complex including the 4 grassland study sites (Figure 2, Table S3), and in 2000–2004 the transects were sampled in each of the 4 grassland and 4 shrubland sites. In 1994–1995, each transect was 2.5 km long and paired with a parallel replicate transect located 1 km away. Each transect had 10 point counts at 250 m intervals (160 point counts total) [56]. In 2000–2004, each transect was 1.2 km long and paired with a parallel replicate transect located 1 km away. Each transect had 5 point counts at 300 m intervals (160 point counts total). For all point counts, the radius was 50 m and sampling time was 5 minutes. The number of individuals of each species and their distance from the center of each point count at 50 m, 100 m and >100 m intervals were recorded.

Small mammal diversity was estimated from 7×7 grids, with 49 Sherman traps each separated by 10 m. In 1992–1996, the sampling took place in two sites in a 15,000 ha colony and one 194 ha colony (Figure 2, Table S3). In each site a grid was established in the interior of the colony, one at the edge, and one in the shrubland 150 m from the edge of the colony. In 2000–2002 the small mammal trapping grids were established in the 4 grassland and 4 shrubland sites (Figure 2, Table S3). Traps were opened for two consecutive nights on each grid. The small mammal trapping grids established on the prairie dog colony grasslands in 2000–2002 were in a different location than those established in 1992–1996 to accommodate different sampling designs.

Lagomorphs and carnivores were sampled along all available roads within the shrubland and prairie dog colony grassland study sites from 1994–1996 and 2000–2002 (Figure 2 and Table S3). Lagomorphs were estimated by spotlighting at night from a vehicle with two 1-million candle searchlights at 8 km/hr, expressed as number of individuals seen per km of transect to standardize transects of variable length [46] (Figure 2, Table S3). During each sighting, species were identified as either desert cottontail or black-tailed jackrabbit and the number of individuals was recorded. Carnivore density was determined on the same road transects, taking the perpendicular distance and angle, with respect to the transect's center, for each carnivore sighted [61].

### Data Analysis

For data analysis of comparisons between vertebrate communities in the shrubland and grassland habitats, we used non-parametric statistics on all data sets, and data for each vertebrate group were pooled across seasons. Statistical significance was set at P<0.05. Wilcoxon Two-Sample (W) tests were used to analyze differences in herpetofauna, birds, and small mammals between the grassland and shrubland habitats [62]. Separate analyses were then conducted for each vertebrate group to evaluate differences in total abundance, species richness, functional groups, trophic groups, and each species between the grassland and shrubland habitats. DCA was used to test for potential differences between the grassland and shrubland sites based on simultaneous analysis of all species of vertebrates [63], [64]. MRPP was used to provide a multivariate test of significance based on Euclidean distances [64], [65]. For the DCA and MRPP, very rare species were removed from the data sets [64], [65]. Chi-square tests then were used to compare the relative abundance of lagomorphs between the grassland and shrubland sites. Carnivore densities were estimated using the program Transect, which compensates for differences in sighting distance between the grassland and shrubland habitats [66]. Given the small sample size, no statistical tests were conducted on the carnivore data. Comparisons were made between prairie dog grasslands over time by calculating the difference in the density of each vertebrate species over time.

## Supporting Information

Table S1 Signs of prairie dog impact on 100 Ephedra trifurca shrubs at the edge of an expanding prairie dog colony, and 50 m away from the colony into the shrubland.
(0.03 MB DOC)

Table S2 Species associated with the prairie dog colony grassland habitat, based on total abundance for herpetofauna, birds, and small mammals and total densities for carnivores over all sample periods in the grassland (Grass) and shurbland (Shrub) habitats. Statistical results for herpetofauna, birds, and small mammals are based on Wilcoxon Two-Sample tests (N = 8). Conservation status in Mexico: SP - Subject to Special Protection, T - Threatened, E - Endangered [67] (SEMARNAT 2002).
(0.06 MB DOC)

Table S3 Sampling periods and effort (in days) for herpetofauna, birds, and small mammals, and kilometers of transect for medium and large mammals in the Janos region of northern Chihuahua.
(0.06 MB DOC)

Figure S1 Mean annual precipitation from 1957–2005 in the Janos-Casas Grandes region. The dotted line indicates the long-term mean annual precipitation for the region (287 mm). The Janos prairie dog colony complex was first mapped in 1988 and then re-mapped in 2005. The vertebrate communities were first sampled in 1994–1996 and then in 2000–2003. Comparisons between the shrubland and grassland communities were made in 2000–2003. The change in land cover was obtained from satellite images from 1990 and 2000.
(0.14 MB DOC)
