# Supplementary material for: Rapid Decline of a Grassland System and Its Ecological and Conservation Implications
Source: PLoS One. 2010 Jan 6;5(1):e8562. doi: 10.1371/journal.pone.0008562 (PMC2797390; doi:10.1371/journal.pone.0008562)
Supplement: Table S1 — Signs of prairie dog impact on 100 Ephedra trifurca shrubs at the edge of an expanding prairie dog colony, and 50 m away from the colony into the shrubland. (0.03 MB DOC) [file pone.0008562.s001.doc]

| **Site** | **Average height (cm)** | **Alive, no impact** | **Dead** | **Teeth marks** | **Stems and branches**  **clipped** | **Burrow in roots** |
| --- | --- | --- | --- | --- | --- | --- |
| Colony edge | 34.16 | 9 | 13 | 7 | 76 | 5 |
| Shrubland | 75.51 | 97 | 0 | 0 | 3 | 0 |
