# Supplementary material for: Rapid Decline of a Grassland System and Its Ecological and Conservation Implications
Source: PLoS One. 2010 Jan 6;5(1):e8562. doi: 10.1371/journal.pone.0008562 (PMC2797390; doi:10.1371/journal.pone.0008562)
Supplement: Table S2 — Species associated with the prairie dog colony grassland habitat, based on total abundance for herpetofauna, birds, and small mammals and total densities for carnivores over all sample periods in the grassland (Grass) and shurbland (Shrub) habitats. Statistical results for herpetofauna, birds, and small mammals are based on Wilcoxon Two-Sample tests (N = 8). Conservation status in Mexico: SP - Subject to Special Protection, T - Threatened, E - Endangered [67] (SEMARNAT 2002). (0.06 MB DOC) [file pone.0008562.s002.doc]

| **Species Name** | **Common Name** | Grass | Shrub | *P* | Status |
| --- | --- | --- | --- | --- | --- |
| HERPETOFAUNA |  |  |  |  |  |
| *Bufo woodhousii* | Woodhouse's Toad | 1 | 0 | 0.3257 |  |
| *Crotalus viridis* | Prairie Rattlesnake | 2 | 0 | 0.1432 | SP |
| *Holbrookia maculata* | Lesser Earless Lizard | 48 | 20 | **0.0197** |  |
| *Spea bombifrons* | Plains Spadefoot Toad | 4 | 0 | 0.1441 |  |
| *Tantilla nigriceps* | Plains Blackhead Snake | 4 | 0 | **0.0481** |  |
|  |  |  |  |  |  |
| BIRDS |  |  |  |  |  |
| *Athene cunicularia* | Burrowing Owl | 138 | 0 | **0.0003** | T |
| *Buteo albonotatus* | Zone-tailed hawk | 1 | 0 | 0.3211 | SP |
| *Buteo regalis* | Ferruginous Hawk | 68 | 3 | **0.0006** | **SP** |
| *Calcarius mccownii* | McCown’s Longspur | 513 | 0 | **0.0484** |  |
| *Dendroica* sp. | Warbler | 1 | 0 | 0.3211 |  |
| *Eremophila alpestris* | Horned Lark | 1446 | 53 | **0.0008** |  |
| *Haliaeetus leucocephalus* | Bald Eagle | 12 | 1 | **0.0295** | E |
| *Numenius americanus* | Long-Billed Curlew | 198 | 0 | **0.0038** |  |
| *Oporornis tolmiei* | MacGillivray’s warbler | 2 | 0 | 0.1589 |  |
| *Sturnella magna* | Eastern Meadowlark | 28 | 0 | **0.0271** |  |
| *Sturnella neglecta* | Western meadowlark | 1 | 0 | 0.3211 |  |
| *Tachycineta bicolor* | Tree Swallow | 15 | 0 | **0.0273** |  |
|  |  |  |  |  |  |
| SMALL MAMMALS |  |  |  |  |  |
| *Baiomys taylori* | Northern Pygmy Mouse | 1 | 0 | 0.3173 |  |
| *Onychomys torridus* | Southern Grasshopper Mouse | 19 | 4 | **0.0362** |  |
| *Perognathus flavus* | Silky Pocket Mouse | 80 | 42 | **0.0348** |  |
|  |  |  |  |  |  |
| CARNIVORES |  |  |  |  |  |
| *Vulpes macrotis* | Kit Fox | 31 | 0 | - | T |
| *Mephitis* spp. | Skunk spp. | 5 | 0 | - |  |
| *Mustela nigripes* | Black-Footed Ferret | 1 | 0 | - | E |
| *Taxidea taxus* | Badger | 2 | 0 | - | T |
