# Supplementary material for: Rapid Decline of a Grassland System and Its Ecological and Conservation Implications
Source: PLoS One. 2010 Jan 6;5(1):e8562. doi: 10.1371/journal.pone.0008562 (PMC2797390; doi:10.1371/journal.pone.0008562)
Supplement: Table S3 — Sampling periods and effort (in days) for herpetofauna, birds, and small mammals, and kilometers of transect for medium and large mammals in the Janos region of northern Chihuahua. (0.06 MB DOC) [file pone.0008562.s003.doc]

| **Month/year** | **Herpetofauna** | **Birds** | **Small mammals** | **Medium/large mammals (km)** |
| --- | --- | --- | --- | --- |
| April, 1992 | 0 | 0 | 9 | 0 |
| May, 1992 | 0 | 0 | 9 | 0 |
| August, 1992 | 0 | 0 | 9 | 0 |
| April, 1993 | 0 | 0 | 12 | 0 |
| May, 1993 | 0 | 0 | 12 | 0 |
| September, 1993 | 0 | 0 | 9 | 0 |
| January, 1994 | 0 | 10 | 0 | 0 |
| April, 1994 | 0 | 0 | 0 | 102 |
| November, 1994 | 0 | 10 | 0 | 0 |
| March, 1995 | 0 | 0 | 0 | 105 |
| May, 1995 | 0 | 10 | 0 | 0 |
| June, 1995 | 0 | 0 | 0 | 104 |
| July, 1995 | 0 | 10 | 0 | 105 |
| August, 1995 | 0 | 0 | 0 | 107 |
| September, 1995 | 0 | 10 | 0 | 91 |
| October, 1995 | 0 | 0 | 0 | 100 |
| November, 1995 | 0 | 10 | 0 | 93 |
| January, 1996 | 0 | 0 | 0 | 90 |
| February, 1996 | 0 | 0 | 0 | 96 |
| March, 1996 | 0 | 0 | 0 | 91 |
| July, 2000 | 0 | 9 | 9 | 80 |
| September, 2000 | 0 | 0 | 8 | 23 |
| November, 2000 | 9 | 8 | 9 | 75 |
| February, 2001 | 0 | 8 | 8 | 73 |
| April, 2001 | 8 | 8 | 8 | 65 |
| July, 2001 | 8 | 0 | 0 | 0 |
| September, 2001 | 8 | 8 | 8 | 59 |
| November, 2001 | 8 | 8 | 8 | 67 |
| June, 2002 | 4 | 9 | 8 | 130 |
| July, 2002 | 8 | 0 | 0 | 0 |
| September, 2002 | 8 | 0 | 0 | 0 |
| February, 2003 | 0 | 7 | 0 | 0 |
| April 2004 | 8 | 0 | 0 | 0 |
| June, 2004 | 0 | 8 | 0 | 0 |
