# Supplementary figures and images for: Rapid Decline of a Grassland System and Its Ecological and Conservation Implications
Source: PLoS One. 2010 Jan 6;5(1):e8562. doi: 10.1371/journal.pone.0008562 (PMC2797390; doi:10.1371/journal.pone.0008562)

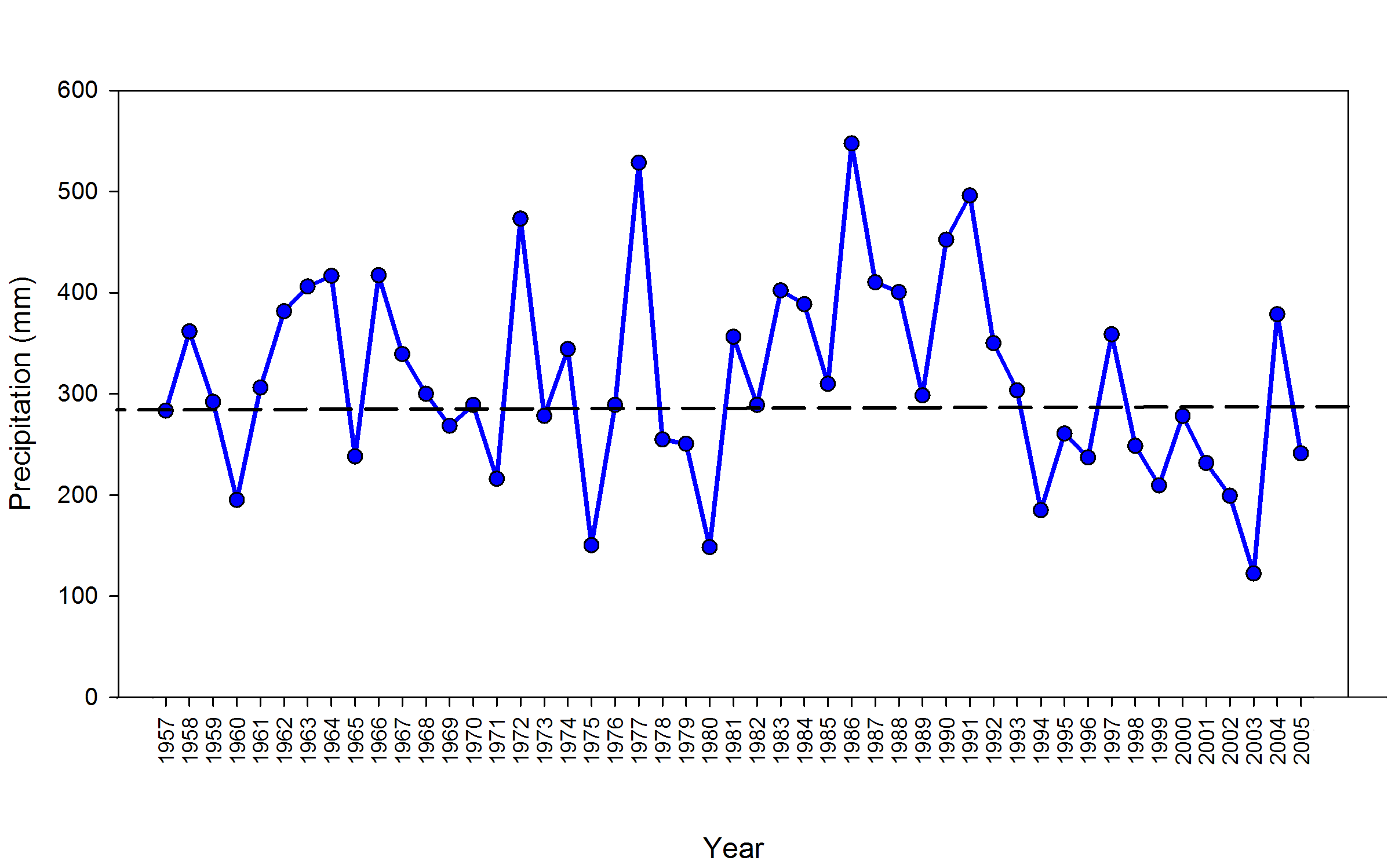

Supplement: Figure S1 — Mean annual precipitation from 1957–2005 in the Janos-Casas Grandes region. The dotted line indicates the long-term mean annual precipitation for the region (287 mm). The Janos prairie dog colony complex was first mapped in 1988 and then re-mapped in 2005. The vertebrate communities were first sampled in 1994–1996 and then in 2000–2003. Comparisons between the shrubland and grassland communities were made in 2000–2003. The change in land cover was obtained from satellite images from 1990 and 2000. (0.14 MB DOC) [file pone.0008562.s004.doc]
